# Superficially located CIC::DUX4-rearranged sarcomas in children: insights from a long-term survival case series

**DOI:** 10.1007/s00428-025-04231-1

**Published:** 2025-10-07

**Authors:** Gina Del Vecchio, Rita Alaggio, Alessandra Stracuzzi, Gabriele Gaggero, Isabella Giovannoni, Sabina Barresi, Sabrina Rossi, Francesca Arienzo, Giuseppe Maria Milano, Ida Russo, Monia Di Prete, Carlo Cota, Maja Cesen, Jessica L. Davis, Daniel Orbach, Damiano Arciuolo

**Affiliations:** 1 Pathology Unit, Bambino Gesù Children’s Hospital, IRCCS, Rome, Italy; 2 Pathology Unit, Giannina Gaslini Institute, IRCCS, Genoa, Italy; 3 Onco-Hematology, Cell Therapy, Gene Therapies and Hemopoietic Transplant, Bambino Gesù Children’s Hospital, IRCCS, Rome, Italy; 4 Dermatopathology Research Unit, San Gallicano Dermatological Institute, IRCCS, Rome, Italy; 5 Department of Pediatric Hematology and Oncology, University of Ljubljana, Ljubljana, Slovenia; 6 Department of Pathology and Laboratory Medicine, Indiana University, Indianapolis, IN USA; 7 SIREDO Oncology Center (Care, Innovation and Research for Children and AYA with Cancer), PSL Research University, Institut Curie, Paris, France; 8 Pathology Institute, Catholic University of Sacred Heart, 00168 Rome, Italy

**Keywords:** Sarcoma small round cell, Sarcoma Ewing-like, CIC-DUX4 fusion oncogene, Pediatric sarcoma, Superficial sarcoma

## Abstract

Soft tissue sarcomas account for approximately 10% of all cancers in the pediatric population, with undifferentiated round cell sarcomas-historically referred to as Ewing-like sarcomas-forming a notable subset. Recent molecular profiling has reclassified these tumors into distinct subtypes, including CIC::DUX4-rearranged sarcomas. While CIC::DUX4 sarcomas are more commonly found in adults, they are rarer in children and are typically located in deep soft tissues. The current study focuses on pediatric cases of superficially located CIC::DUX4 sarcomas, aiming to describe their clinical, morphological, and molecular features. This retrospective study includes pediatric patients diagnosed with superficial CIC::DUX4-rearranged sarcoma from January 2018 to December 2024. Relevant clinical data, including patient demographics, tumor location, and follow-up outcomes, were extracted from medical records. Tumor samples were examined histologically, and RNA sequencing was performed to confirm the presence of the CIC::DUX4 fusion. For survival analysis, we compared our series with 27 pediatric deep-seated CIC::DUX4 sarcomas. Five cases of superficial CIC::DUX4 sarcoma were identified. The patients ranged from 4 to 18 years old, with a mean age of 10.6 years. Only one patient presented with pulmonary metastases at diagnosis. All patients underwent excisional biopsies. Follow-up revealed complete remission in all cases and a statistically significant difference in OS (*p* = 0.00256) and DFS (*p* = 0.0239) compared to deep-seated CIC::DUX4 sarcoma. This study presents the first pediatric case series of superficial CIC::DUX4 sarcomas. While CIC::DUX4 sarcomas in adults are known for their aggressive behavior, our findings suggest that superficial tumors with well-defined margins in children may have a more favorable prognosis. These results highlight the need for further research into the biological behavior and long-term outcomes of these rare pediatric tumors.

## Introduction

Soft tissue sarcomas represent approximately 10% of all cancers in the pediatric population, making them a relevant category of malignancies in this age group [1]. Within this category, undifferentiated round cell sarcomas—historically referred to as Ewing-like sarcomas—form a distinctive subset of tumors that primarily affect children and adolescents. These tumors exhibit morphological similarities to Ewing sarcoma but are distinguished by the absence of the characteristic fusions between the EWSR1 gene and members of the ETS transcription factor family, such as FLI1 or ERG, which define classical Ewing sarcoma [2].

Over the past decade, the application of advanced molecular diagnostic techniques has significantly refined the classification of these tumors. According to the latest WHO classification of soft tissue and bone tumors, undifferentiated round cell sarcomas, besides Ewing sarcoma, are now divided into three molecularly distinct subcategories: round cell sarcomas with EWSR1 fusions involving non-ETS partners, CIC-rearranged sarcomas, and BCOR-rearranged sarcomas [2].

This reclassification underscores the growing importance of molecular profiling in guiding the diagnosis and management of these tumors, as their clinical behavior, prognosis, and therapeutic responses can vary considerably.

Among these entities, the CIC::DUX4 gene fusion is the most prevalent genetic alteration identified in EWSR1-negative adults undifferentiated round cell sarcomas, while it is rarer in children, in which BCOR::CCNB3 and other BCOR alterations are more frequent.

CIC::DUX4 results from t(4;19)(q35;q13) or t(10;19)(q26;q13) chromosomal translocations and involves two key genes: CIC, a transcriptional repressor located on chromosome 19q13.1, which plays a role in regulating gene expression during normal cellular processes, and DUX4, a double homeobox transcription factor situated on chromosome 4q35 or 10q26.3 [3].

The resulting CIC::DUX4 fusion leads to aberrant gene expression profiles that drive tumorigenesis.

CIC::DUX4 sarcomas most frequently involve the deep soft tissues of the head, neck, retroperitoneum, and pelvis, but they can occasionally affect visceral organs or bones [2]. Clinical outcome is generally poor, with a reported 3-year overall survival rate of approximately 44–46% [4, 5]. Metastatic disease is commonly observed at diagnosis, further complicating management and contributing to the unfavorable prognosis.

Interestingly, superficial occurrences of CIC::DUX4 sarcomas are exceedingly rare. Only a limited number of cases have been described in the literature, and these have shown similar histological features and clinical outcomes to their deep-seated counterparts [6–9]. Reports of superficially located cases are sporadic and primarily involve adult patients, while only two cases have been described in the literature in the pediatric group [9, 10].

In this context, superficial refers to tumors located entirely above the superficial fascia, without involvement of the underlying muscular or deep soft tissue compartments. This anatomical definition is clinically relevant, as superficial soft tissue sarcomas may exhibit distinct biological behavior and are often associated with a more favorable prognosis compared to their deep-seated counterparts. A similar prognostic distinction has been reported in superficially located Ewing sarcomas, which tend to show improved clinical outcomes compared to deeply situated lesions [11]. In light of these observations, the present study aims to provide insights into the clinical behavior and outcome of superficially located CIC::DUX4 undifferentiated sarcomas by analyzing a series of pediatric cases with detailed clinical, morphological, and molecular profiling.

## Materials and methods

### Case selection

A series of consecutive superficially located CIC::DUX sarcoma diagnosed in patients up to 18 years of age was included in the study. These cases were identified from the institutional files of the institution involved in the study and from consultation records spanning the period from January 2018 to December 2024.

Relevant clinical data, including patient age, gender, tumor location, and follow-up information, were retrieved from medical records and pathology reports. The clinical details were reviewed and verified to ensure accuracy and completeness.

This study was conducted in full compliance with the ethical standards outlined by the institutional ethics committee of the participating institutions. Written informed consent was obtained from the parents or legal guardians of each patient prior to the inclusion of their data in this research. The informed consent process adhered to all applicable national and international regulations governing pediatric research.

All the available histologic slides and immunostains were reviewed by a panel of expert pediatric pathologists.

For comparative purposes, we also analyzed a previously published cohort of deep-seated CIC::DUX4-rearranged sarcomas [4]. To ensure consistency with our inclusion criteria, we excluded cases involving patients older than 18 years and supplemented the series with two additional deep-seated cases from our institutional files that became available during the study period, resulting in a total of 27 pediatric deep-seated CIC::DUX4-rearranged sarcomas.

### RNA sequencing

Total RNA was extracted from FFPE sections of the tumor using Maxwell CSC FFPE RNA Extraction Kit (Promega) for the automatic extraction.

Two hundred nanograms of RNA was used for library preparation with the Archer Custom Fusion Plex Kit (ArcherDX, CO), according to the manufacturer’s protocols. RNA libraries were pooled in equimolar amounts of 4 nM (10 libraries/pool) and loaded at 10 pM with MiSeq Reagent Kits v3 600 cycles on MiSEQ platform (Illumina, San Diego, California). NGS data were analyzed using Archer Data Analysis Software v6.2.3. For Case 4, the library for RNA sequencing was prepared using the SureSelect XT HS2 (Agilent Technologies) kit following the manufacturer’s instructions. The sequencing run was performed in paired-end mode (2 × 151-bp reads) using the Illumina NextSeQ 550 platform. Raw reads were aligned to the reference human genome (UCSC-Build38) using STAR (2.5.3a) algorithm, and candidate fusion transcripts identification was then performed using Arriba Fusion pipeline [12].

### Statistical analysis

Follow-up duration was summarized using the median and interquartile range (IQR). Survival curves for disease-free survival (DFS) and overall survival (OS) were estimated using the Kaplan–Meier method and compared between groups using the log-rank (Mantel–Cox) test. A *p*-value < 0.05 was considered statistically significant. All statistical analyses and graphical representations were performed using GraphPad Prism software 10.5.0 (774).

## Results

Five superficial undifferentiated sarcomas harboring CIC::DUX4 fusion were identified (Table 1). The precise anatomical site of the lesions was reported in four out of five cases: one in the plantar region, one in the supraclavicular region of the shoulder, and two in the hand. The remaining case exhibited dermal involvement; however, the exact site was not specified.

**Table 1:** Clinical and molecular features of the superficial CIC::DUX4- rearranged sarcoma

Patient	Site	Molecular technique	Breakpoint of CIC	Breakpoint of DUX4	DUX4 IHC
1	Superficial	Archer fusion panel	Exon 20	Exon 1	NA
2	Superficial	Archer fusion panel	Exon 20	Exon 1	NA
3	Superficial	Archer fusion panel	Exon 20	Exon 1	NA
4	Superficial	RNA-Seq	Exon 15	Exon 1	NA
5	Superficial	NA	NA	NA	Positive

*NA* not available

The patients were 4, 7, 11, 13, and 18 years old at the time of diagnosis, with a mean age of 10.6 years (Table 2). The cohort consisted of four male patients (4, 7, 13, and 18 years old) and one female patient (11 years old). Remarkably, the 4-year-old patient came to our attention when pulmonary metastases were already present; however, his clinical history began at the age of two when the same lesion was clinically diagnosed as a vascular malformation based on imaging. None of the remaining patients had metastatic disease at the time of diagnosis. Follow-up data, available for all cases, revealed complete remission at 29, 69, 16, 10, and 30 months. Excisional biopsies were performed in all cases. In two cases, positive surgical margins necessitated a reintervention to achieve complete excision. Moreover, all patients—except for case number 17 (Table 2)—received adjuvant chemotherapy with the VDC/IE regimen (vincristine, doxorubicin, and cyclophosphamide alternating with ifosfamide and etoposide). No radiotherapy was administered.

**Table 2:** Clinical features of superficially located CIC::DUX4 sarcoma, including our cases and all other cases previously reported in the literature

	Patient	Age (y)	Sex	Site	Size (mm)	Recurrence/metastasis	Follow-up (months)
Lehane (2019)	1	28	F	Forearm	15	Absent	NED (18)
Bricic (2020)	2	35	F	Ear	Unknown	Absent	NED (24)
Ko (2020)	3	14	F	Vulva	40	Lung	DOD (23)
	4	65	M	Forearm	30	Recurrence	AWD (48)
	5	55	F	Hand	80	Lung and LN	DOD (24)
	6	32	F	Foot	Unknown	LN	DOD (22)
	7	58	F	Labium	5	Absent	NED (12)
	8	26	F	Flank	20	Absent	NED (2)
	9	35	F	Lower flank	10	Unknown	Unknown
	10	55	M	Lower leg	20	Unknown	Unknown
Maloney (2020)	11	55	F	Shoulder	Unknown	Unknown	Unknown
	12	50	M	Thigh	30	Recurrence + lung	Unknown
	13	27	F	Leg	Unknown	Absent	NED (36)
Maejima (2021)	14	14	M	Occipital	60	Absent	NED (14)
Faden (2024)	15	23	M	Buttock	110	Absent	NED (14)
Present case series	16	4	M	Plantar	30	Lung	NED (309)
	17	7	M	Supraclavicular	23	Absent	NED (70)
	18	11	F	Hand	16	Absent	NED (17)
	19	13	M	Unknown	22	Absent	NED (11)
	20	18	M	Hand	12	Absent	NED (31)

*NED* no evidence of disease, *DOD* dead of disease, *LN* lymph node

### Histopathological features

The five lesions, measuring between 11 and 23 mm, were dermal to subcutaneous based. Three of them (Cases 2, 3, and 4) appeared well-circumscribed, while two cases (Cases 1 and 5) exhibited focal infiltrative growth.

**Fig. 1:**
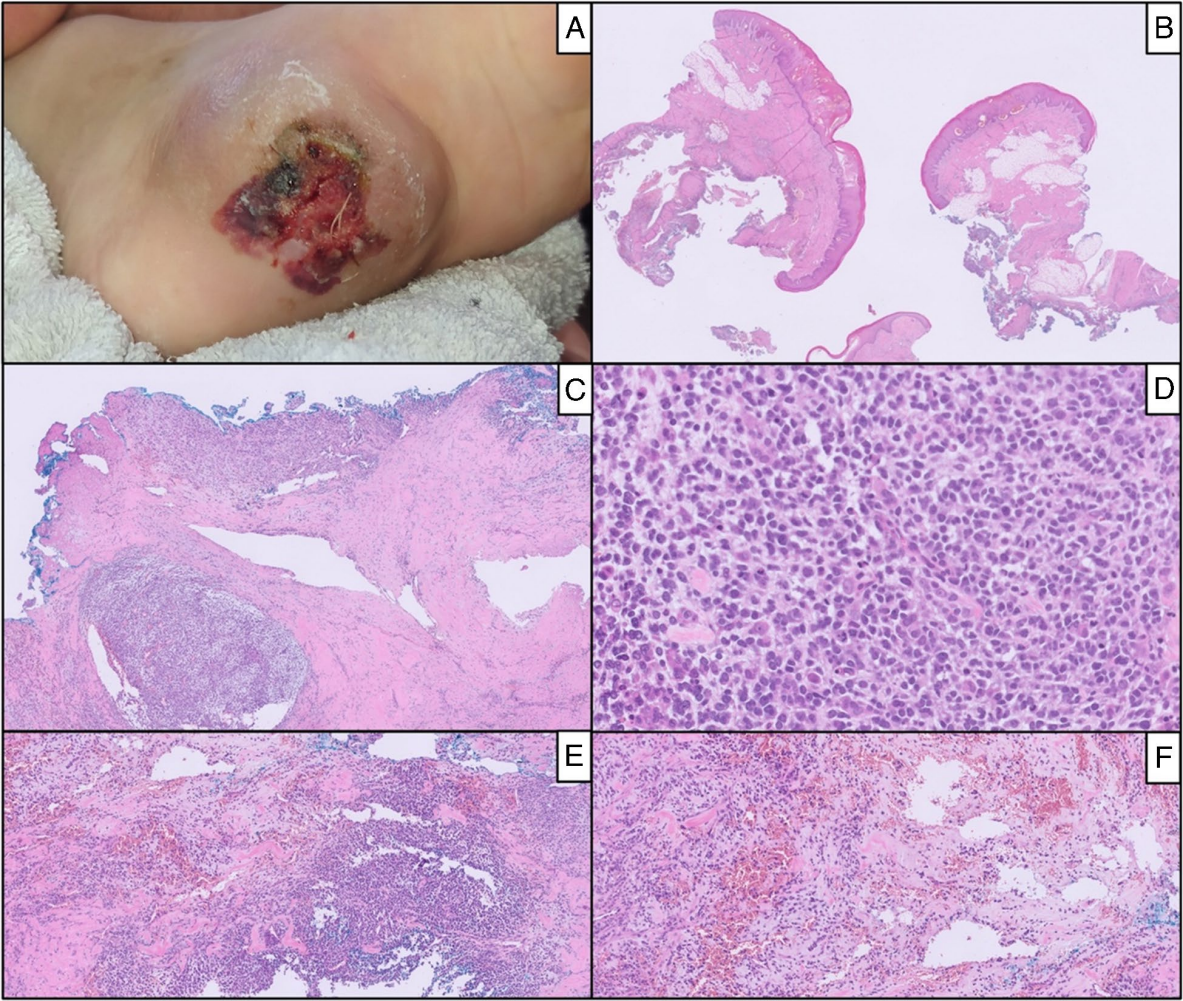
Case 1: Ulcerated lesion (**A**) infiltrating the deep dermis and adipose tissue (**B** and **C**), composed of epithelioid cells in a fibro-myxoid stroma (**D**) accompanied—at the periphery—by pseudovascular spaces (**E** and **F**)

**Fig. 2:**
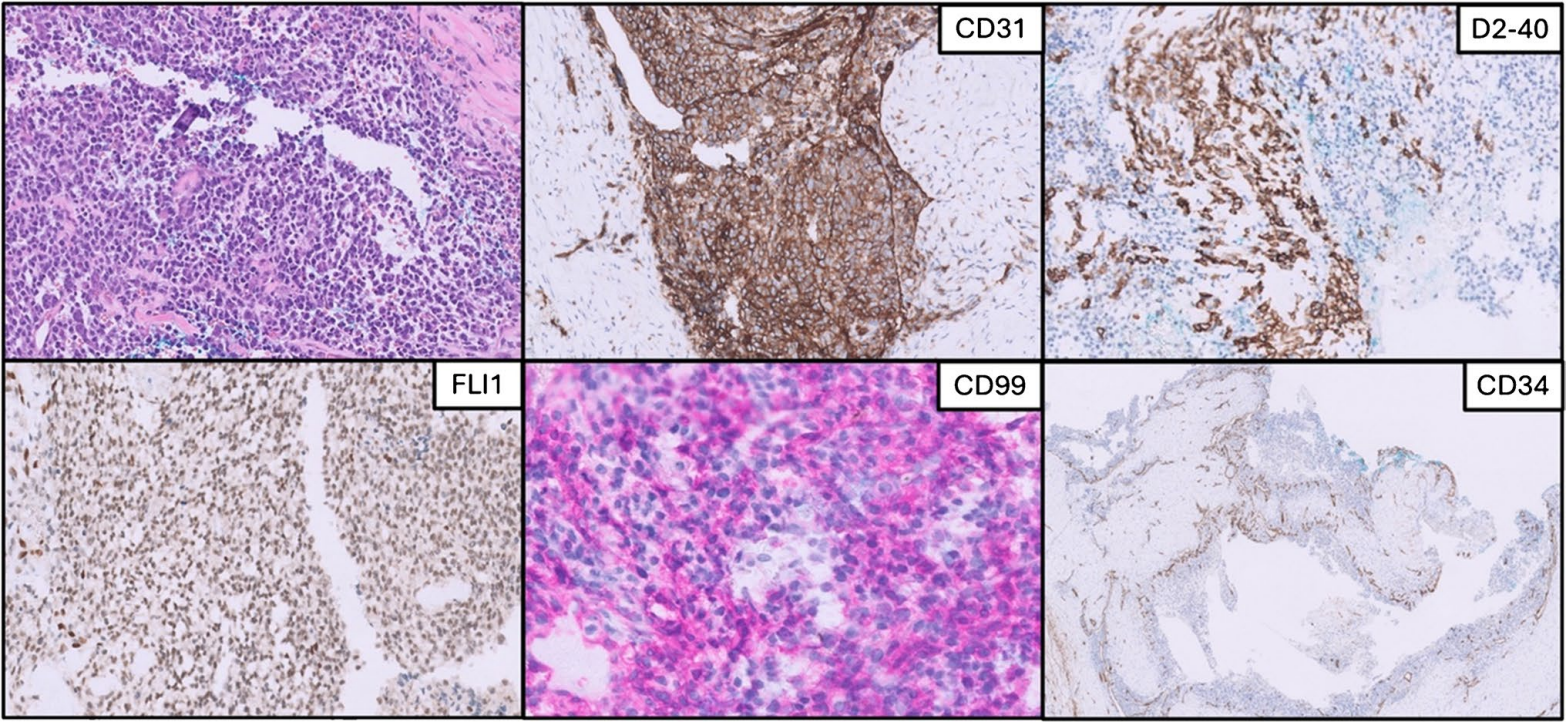
Case 1: Solid and pseudovascular areas with positive immunostainings for CD31, D2-40, FL11, CD99, and negative CD34

**Fig. 3:**
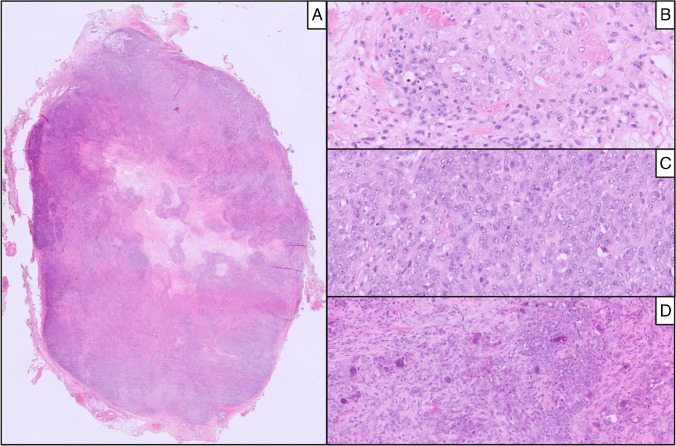
Case 3: A well-circumscribed lesion with pushing margin and central necrosis (**A**) composed of epithelioid cells exhibiting clear to eosinophilic cytoplasm (**B**) and high mitotic activity (**C**), coexisting with numerous osteoclast-like giant cells

**Fig. 4:**
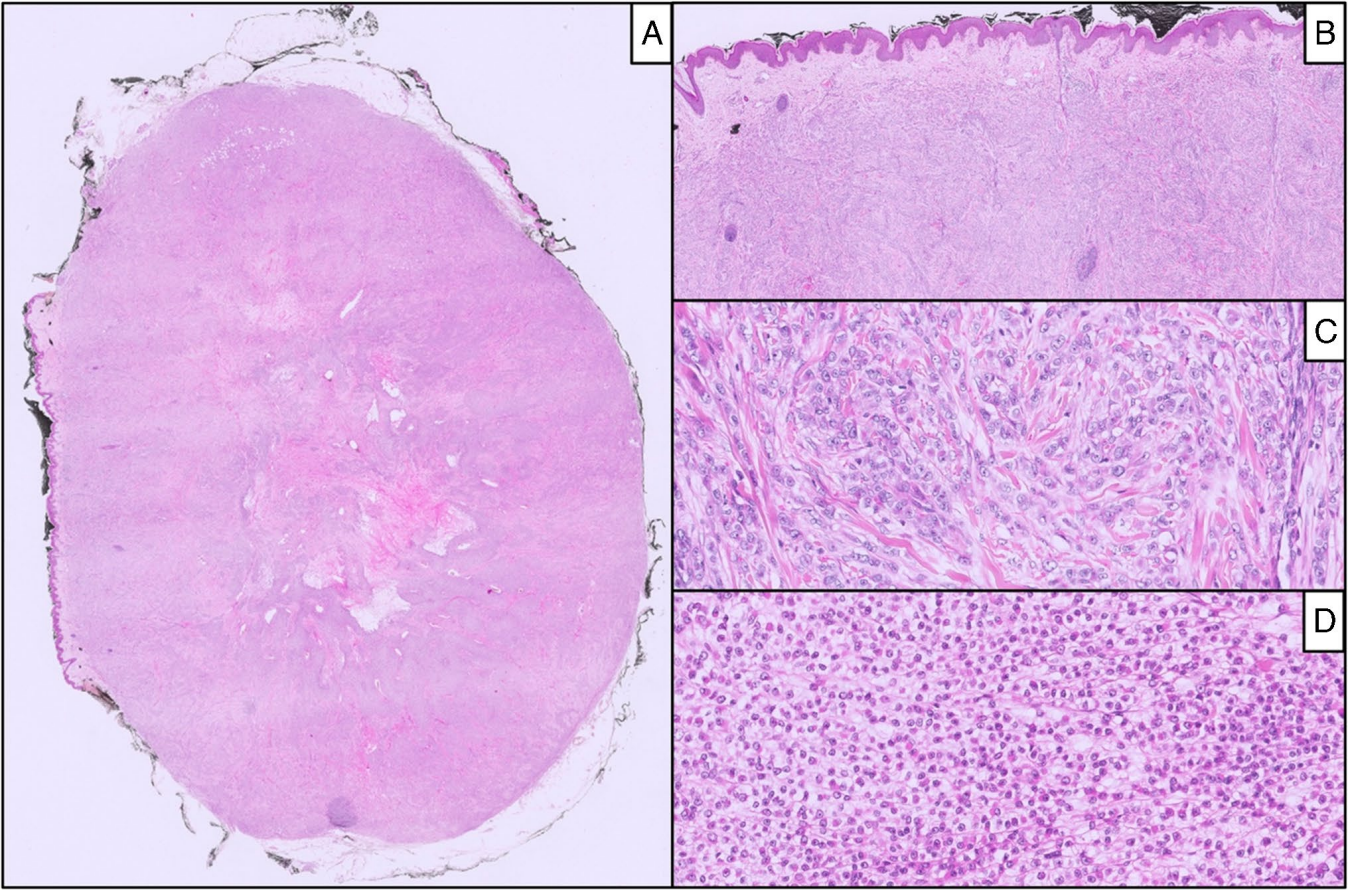
Case 4: At low-power field, a nodular lesion with pushing margin (**A**), located in the dermis (**B**) and composed of epithelioid cells with moderate, clear cytoplasm organized in nest and sheet (**C** and **D**)

Histologically (see Figs. 1, 2, 3, and 4), all tumors were composed of medium-sized cells with epithelioid to spindle or round morphology. The neoplastic cells showed oval to round nuclei with vesicular chromatin and prominent eosinophilic nucleoli. The cytoplasm varied from eosinophilic to clear across the cases. In Case 5, a vague rhabdoid appearance was observed, whereas Case 3 featured numerous osteoclast-like giant cells. The background stroma was predominantly fibrous or collagenized in all cases, with focal areas of myxoid or chondro-myxoid differentiation in three (Cases 1, 2, and 5). Thin-walled and occasionally dilated vessels were consistently present. Pseudo-vascular spaces lined by tumor cells were identified in Cases 1, 2, and 4.

Foci of necrosis were found in four cases (Cases 1, 2, 4, and 5), and perineural infiltration was seen in Case 3. A lymphoid or mixed inflammatory infiltrate was observed in three cases (Cases 1, 2, and 4). Mitotic activity ranged from 2 to 8 mitoses per ten high-power fields across the first four cases, while Case 5 demonstrated markedly increased proliferative activity, with numerous mitotic figures, particularly in the more densely cellular areas.

Immunohistochemically, CD99 was diffusely positive in all five cases. CD31 was positive in Cases 1, 2, and 3, while ERG showed focal nuclear positivity in Cases 1 and 5, but was negative in Cases 3 and 4. FLI1 demonstrated focal nuclear staining in Cases 1 and 2. PROX1 and D2-40 were positive in Case 1. WT1 expression was seen in Cases 2 and 5. Case 5 showed diffuse nuclear positivity for DUX4 and retained INI1 expression. CD163 was focally positive in Case 2. All tested cases were negative for CD34, epithelial markers (CK, EMA), melanocytic markers (S100, SOX10), myogenic markers (desmin, calponin), and other lineage markers including CCNB3 and p63.

### Clinical features

**Fig. 5:**
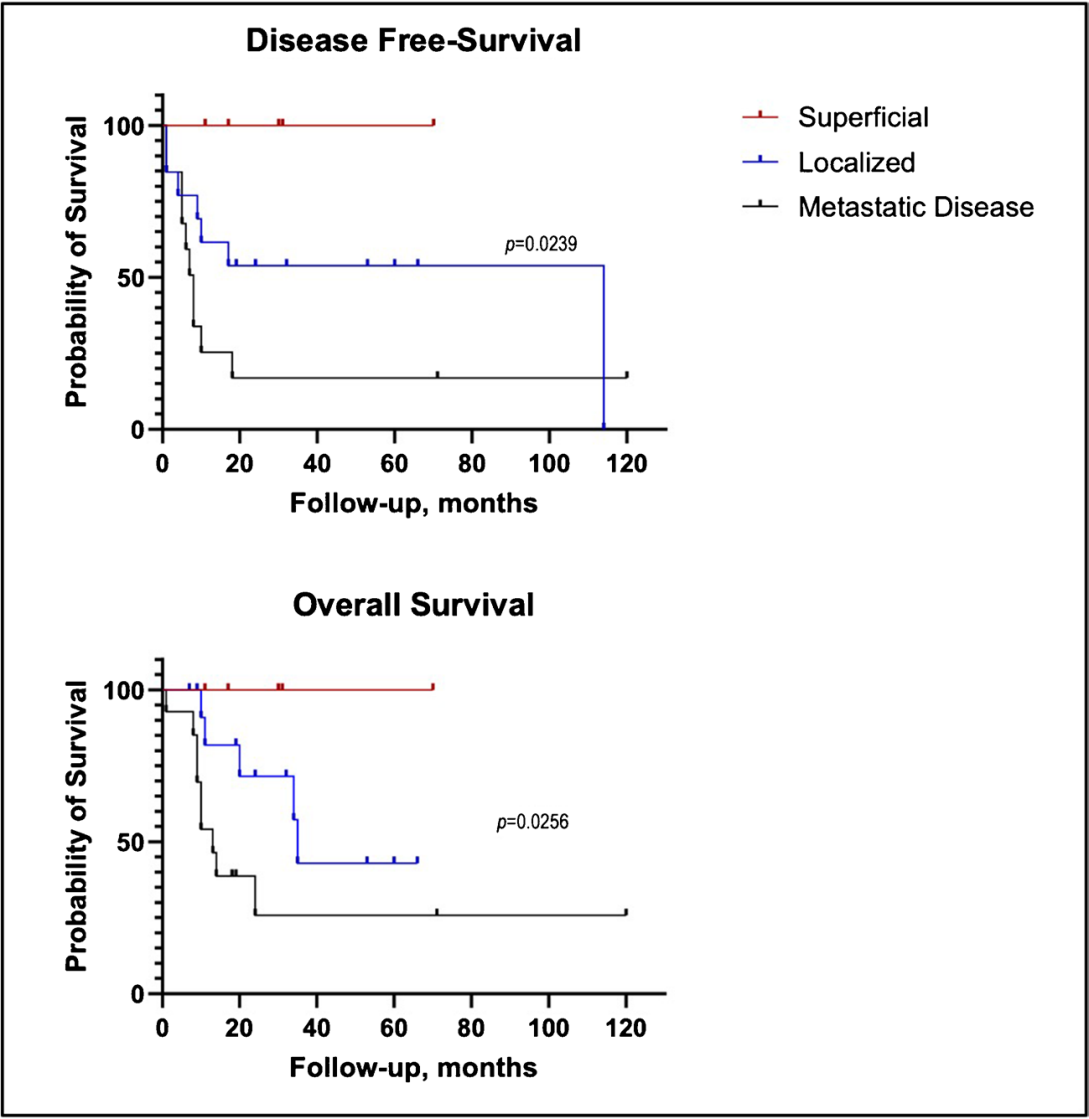
Kaplan–Meier curves for disease-free survival (DFS, top panel) and overall survival (OS, bottom panel) in 32 pediatric patients with CIC::DUX4-rearranged sarcomas, stratified by clinical presentation: superficial (red) and deep-seated, localized (blue), and metastatic at diagnosis (black). A statistically significant difference was observed among the three groups for both DFS (*p* = 0.0239) and OS (*p* = 0.0256), with superficial cases showing markedly better outcomes and no events during follow-up

Our series includes 32 pediatric CIC::DUX4 rearranged sarcoma, 5 superficial and 27 deep-seated (Table 3), with a median follow-up of 18.5 months (IQR, 10.0–33.5). The median follow-up was 30.0 months (IQR: 14.0–50.5) for superficial CIC::DUX4 sarcomas, 14.0 months (IQR: 9.0–24.0) for localized deep-seated CIC::DUX4 sarcomas (n = 13), and 19.5 months (IQR: 9.75–39.5) for those deep-seated metastatic cases at diagnosis (n = 14). Kaplan–Meier survival analysis using the log-rank test revealed a statistically significant difference in DFS and OS between the groups (*p* = 0.0239, *p* = 0.0256, respectively; see Fig. 5).

**Table 3:** Clinical features of deep-seated CIC::DUX4 sarcoma

Patient	Age (y)	Sex	Metastatic at diagnosis/site	Recurrence/metastasis (months)	Follow-up status (months)
1	12	M	No	No	NED (66)
2	17	F	Yes/lung	No	NED (71)
3	11	F	Yes/lung	No	NED (1)
4	14	M	Yes/lung	No	NED (120)
5	13	M	Yes/lung	Yes (6)	DOD (24)
6	17	F	No	Yes (9)	DOD (10)
7	13	F	No	Yes (10)	DOD (11)
8	18		No	No	NED (53)
9	16	M	Yes/lung	Yes (7)	DOD (13)
10	18		No	No	NED (24)
11	3	F	No	Yes (114)	DOD (35)
12	11	F	Yes/lung	No	NED (18)
13	14	M	Yes/peritoneum	Yes (0)	DOD (19)
14	16	F	Yes/lung	Yes (8)	DOD (9)
15	15	M	Yes/peritoneum	Yes (8)	DOD (10)
16	3	F	No	Yes (4)	DOD (34)
17	16	M	Yes/peritoneum	Yes (1)	DOD (8)
18	17	F	Yes/unknown	Yes (5)	DOD (9)
19	11	F	No	Yes (1)	NED (32)
20	18	F	No	No	NED (7)
21	1	F	No	Yes (1)	NED (9)
22	12	M	No	No	NED (19)
23	13	M	Yes/lung	Yes (19)	NED (19)
24	13	M	No	No	NED (60)
25	13	F	No	Yes (17)	DOD (20)
26	17	F	No	Yes (8)	DOD (8)
27	18	F	Yes/lung	Yes (1)	DOD (1)

*NED* no evidence of disease, *DOD* dead of disease

As expected, metastatic disease was associated with a worse prognosis. Eight out of 14 metastatic patients experienced local and/or distant progression, and nine patients died during follow-up. The estimated DFS and OS rates at 12 months were 36.9% and 46.4%, respectively, dropping to 24.6% and 25.8% at 24 months.

Localized tumors also had a relatively poor prognosis compared to superficial cases. Disease progression occurred in seven of 13 patients, and five died during follow-up. Disease progression occurred in seven of 13 patients, and five died during follow-up. The 24-month DFS and OS rates were 53.8% and 71.6%, respectively, with OS declining to 42.9% at 5 years.

In the superficial group, no cases showed disease progression and are all alive and well.

## Discussion

Herein, we reported the first case series of superficial CIC::DUX4 sarcomas in children. CIC::DUX4-rearranged sarcomas represent a relatively recently described group of high-grade neoplasms, characterized by the fusion of the CIC gene with the DUX4 gene [2]. The CIC gene is the human homolog of the *Drosophila melanogaster* capicua gene, encoding a transcriptional repressor [13], while the DUX4 gene encodes the double homeobox 4 protein, whose normal function remains poorly understood [14]. However, DUX4 is known to be normally expressed in germ cells and epigenetically silenced in somatic differentiated tissues [15]. Its aberrant expression in various cancers suggests its potential role in generating a cancer cell population resembling early embryonic stem cells [16].

The morphological and immunophenotypical features observed in our cases mirrored those of classic CIC::DUX4 sarcomas typically encountered in deeper anatomical locations [2, 15]. Intriguingly, a pseudovascular pattern was seen in a subset of cases (2/5 cases). The immunopositivity for endothelial markers (e.g., CD31, ERG, and FLI1) can be a diagnostic challenge, mimicking an epithelioid angiosarcoma [9, 17]. Indeed, these findings have been highlighted in previous studies leading to suspect a potential vascular differentiation in CIC::DUX4 sarcomas. Moreover, CIC::DUX4 sarcomas can sometimes mimic the morphological features of angiosarcomas by forming pseudovascular spaces and cellular arrangements resembling angiosarcomas. It is thought that the expression of endothelial markers is related to a transcriptional deregulation induced by the CIC::DUX4 fusion. This fusion protein likely generates a dysregulated transcription factor, upregulating a spectrum of target genes, including ETS family proteins, which may drive endothelial marker expression [18].

To further complicate the differential diagnosis, Huang et al. [19] reported a subset of non-vasoformative angiosarcoma with CIC rearrangements. Thus, while CIC alterations have been observed in tumors with angiosarcoma-like features, this contributes to the diagnostic challenges in distinguishing between true vascular neoplasms and undifferentiated sarcomas with aberrant endothelial marker expression.

The heterogeneous morphology of CIC::DUX4 sarcomas and the challenge of establishing a diagnosis based solely on histological evaluation without molecular confirmation are amplified by Case 3, which displayed numerous multinucleated giant cells. While the presence of multinucleated giant cells has been occasionally reported in rare cases of CIC::DUX4-rearranged undifferentiated small cell sarcomas [20], this case further expands the recognized morphologic spectrum of such tumors in pediatric patients. The differential diagnosis includes other malignant mesenchymal neoplasms characterized by a prominent giant cell component. However, the immunohistochemical and molecular findings in the current case effectively excluded other giant cell–rich neoplasms. Instead, the morphologic and molecular findings reinforce the notion that CIC::DUX4 sarcomas can exhibit a broader histologic heterogeneity than previously appreciated, underscoring the necessity of molecular testing for accurate classification.

From a clinical standpoint, CIC::DUX4 sarcomas, regardless of location, generally exhibit a poorer prognosis compared to the Ewing sarcoma and other “Ewing-like” sarcomas [4, 5, 21]. While our series has a small number of cases and therefore does not allow us to draw a definitive conclusion, it does highlight a longer survival in children affected by superficial CIC::DUX4 sarcomas. In fact, considering our cases, together with the two pediatric cases reported in the literature, all patients remain in complete remission, except one which was primarily metastatic at the time of diagnosis (Case 3, Table 2) [9]. Although, it can be argued that in one patient, in the current series (case 19, Table 2), the follow-up is too short (11 months), the remaining patients have a follow-up ranging between 17 and 70 months. It is also noteworthy that Case 1 (patient 16, Table 2), despite a prolonged clinical history and the presence of lung metastases at diagnosis, is still alive and without evidence of disease at 30 months—an outcome that stands in marked contrast to the typically aggressive clinical course observed in most CIC::DUX4 cases. This observation gains further relevance when compared with published clinical outcomes in CIC sarcoma patients, where the disease typically follows an aggressive course. In one such study, the mean overall survival (OS) was 22.1 months, and the mean progression-free survival (PFS) was 16.7 months. Notably, among patients presenting with localized disease, progression occurred in two cases—one with local recurrence 5.3 months after diagnosis and one with distant metastases at 10 months—both resulting in death, with a mean OS of 19.3 months. In patients who presented with metastatic disease at diagnosis, the mean PFS was 9.7 months, and the mean OS was 16.3 months. These findings indicate that disease progression typically occurs within the first year; therefore, although limited, the follow-up duration available for most patients in our series appears sufficient to capture the critical window in which recurrences and metastases are most likely to occur. The absence of such events in our cohort may reflect a distinct clinical behavior in pediatric superficial cases, which warrants further investigation in larger, dedicated studies [22].

Finally, Maloney et al. [7], in their series, highlighted the potential prognostic relevance of well-circumscribed margins independently from age. This was based on one of their three patients who remained disease-free 36 months after excision and morphologically exhibited a well-circumscribed tumor. This is also supported by the evidence of a circumscribed growth pattern in three out of five cases in our series.

In conclusion, the cases described in this series underscore the potential for a more favorable clinical course in superficial CIC::DUX in pediatric patients. Although the number of cases is limited, the current series highlights the need for international cooperative studies to identify potential prognostic categories in these rare entities in order to better define their biology and address future therapeutic strategies.
